# Trade-Off and Projecting Effects of Land Use Change on Ecosystem Services under Different Policies Scenarios: A Case Study in Central China

**DOI:** 10.3390/ijerph18073552

**Published:** 2021-03-29

**Authors:** Zhengxin Ji, Hejie Wei, Dong Xue, Mengxue Liu, Enxiang Cai, Weiqiang Chen, Xinwei Feng, Jiwei Li, Jie Lu, Yulong Guo

**Affiliations:** 1 College of Resources and Environmental Sciences, Henan Agricultural University, Zhengzhou 450002, China; S20193030520@cau.edu.cn (Z.J.); a18837158453@163.com (D.X.); caiex213@henau.edu.cn (E.C.); chwqgis@henau.edu.cn (W.C.); xwfeng@henau.edu.cn (X.F.); lijiwei@henau.edu.cn (J.L.); lujie706@henau.edu.cn (J.L.); gyl.zh@henau.edu.cn (Y.G.); 2 Henan Engineering Research Center of Land Consolidation and Ecological Restoration, Henan Agricultural University, Zhengzhou 450002, China; 3 Faculty of Geographical Science, Beijing Normal University, Beijing 100875, China; mengxueliu@mail.bnu.edu.cn

**Keywords:** ecosystem service value, land use change, scenario simulation, trade-offs and synergies, rapidly urbanized area

## Abstract

Predicting the spatio-temporal evolution characteristics and trade-off/synergy relationships of ecosystem service value (ESV) under different policy scenarios is of great significance for realizing regional sustainable development. This study established a framework and used the geographical simulation and optimization systems-future land use simulation (GeoSOS-FLUS) model and bivariate local autocorrelation analysis to stimulate and predict the impact of land use change on the ESV of Anyang City from 1995 to 2025. We also explored the trade-offs and synergy among ecosystem services under three policy scenarios (natural evolution, cultivated land protection, and ecological protection) in 2025. Results show that (1) the land use change in Anyang from 1995 to 2025 was significant, and the degree of land use change under the cultivated land and ecological protection scenarios was more moderate than that under the natural evolution scenario; (2) The total ESV decreased between 1995 and 2015, amounting to losses of 1126 million yuan, and the decline from 2015 to 2025 under the natural evolution scenario was more significant than those under the cultivated land protection and ecological protection scenarios; and (3) an obvious synergy was observed between various ecosystem services in Anyang City under different scenarios in 2025, and the most significant synergy was observed under the natural evolution scenario. In terms of spatial distribution, the agglomeration of “high–high” synergy in the west and “low–low” synergy in the central region was significant. Local areas showed “high–low” and “low–high” trade-off relationships scattered between their built land and woodland or cultivated land. The proposed framework can provide certain scientific support for regulating land use and ecosystem services in rapidly urbanized areas.

## 1. Introduction

Ecosystem services are products (such as food and energy) or services (such as air quality regulation) that humans can obtain directly or indirectly from the ecosystem to meet their needs for survival, health, and well-being [1,2,3]. Over the past 50 years, 63% of the global ecosystem services have been seriously attenuated, and human activities are among the key causes [4]. Land use change is the most direct response and manifestation of natural ecosystems to human activities [5,6,7,8]. Based on different social development needs, human beings create different land use patterns on the land surface, thereby driving changes in regional land use types and in the structure, process, and function of the ecosystem [9,10,11]. These changes are reflected and characterized through the evolution of ecosystem service value (ESV) [12,13,14]. In recent years, as some international projects such as the global land project, the economics of ecosystems and biodiversity, and the intergovernmental platform on biodiversity and ecosystem services have been implemented, the integration of land use/cover change and ESV has become a very popular research topic [15,16,17,18,19]. A quantitative assessment of ESV from the land use change perspective has also become a core issue related to global environmental changes [20].

Since reform and opening up in 1978, the rapid economic development and urbanization process in China have greatly promoted the rapid transformation of land use around its cities [21], thereby leading to a series of problems, such as the loss of major ecosystem services including food production [22], reduced biodiversity [23,24], and poor environmental quality [25], all of which have changed the direction and magnitude of regional ESV [26,27]. Therefore, the impact of land use changes caused by urban expansion on ESV has attracted academic attention [28,29,30,31]. However, ESV is spatially heterogeneous, and previous case studies and conclusions cannot be easily extended to other areas experiencing rapid urbanization [32]. At the same time, many of these studies have focused on the impact of spatial and temporal changes in urban land use/cover on ESV without predicting and simulating future ESV and investigating the trade-off/synergy relationship among ecosystem services under different land use change scenarios [33,34,35]. Specifically, spatial visualization research on the balance/collaboration of ecosystem services remains insufficient. In view of the complexity and uncertainty of future land use changes in rapidly urbanized regions, multi-scenario simulation and analysis should be conducted to assess the future impact of land use change on ESV under different policy scenarios and to understand the trade-off and synergy among ecosystem services in order to provide a scientific basis for regional natural resource management, policy formulation, and sustainable ecosystem development.

Anyang is a regional central city located at the junction of Henan, Shanxi, and Hebei and is a core city in the coordinated development area around Beijing, Tianjin, and Hebei. Following the advancement of the Beijing–Tianjin–Hebei integration, the implementation of the coordination and linkage strategy of the surrounding regions, the commencement of the “Belt and Road Initiative,” and the rapid formation of the Central Plains economic region, Anyang has become a typical rapidly urbanized area in Central China that shows dramatic changes in its land use patterns, complex structure and function, and rapid economic and social development. Anyang is also placed at the core of ecological protection and high-quality development planning in the Yellow River Basin and is considered an important area for water and soil conservation in the national ecological function zoning. In sum, the ecological strategic position of this city has great significance. The rapid economic development and urbanization of Anyang have led to an increasingly intensified regional competition for different land use types, and the contradiction between the demands of social and economic development and the pressure of ecological environmental protection has become increasingly significant. Therefore, exploring the spatio-temporal evolution characteristics of the ESV of Anyang and predicting the impact of land use changes under different scenarios can contribute to making optimized land use decisions and coordinating land spatial patterns. By analyzing the spatio-temporal evolution characteristics of land use and the geographical distribution of the ESV of Anyang between 1995 and 2015, this study developed the geographical simulation and optimization systems-future land use simulation (GeoSOS-FLUS) model for analyzing the impact of land use change on the ESV of Anyang in 2025 under different scenarios. Spatial autocorrelation was performed to explore the trade-offs and synergy among various ecosystem services in Anyang to provide scientific support for the construction of an ecological security pattern and to promote a coordinated development among the social economies of Anyang and other similar rapidly urbanized regions.

## 2. Materials and Methods

### 2.1. The Study Area

**Figure 1:**
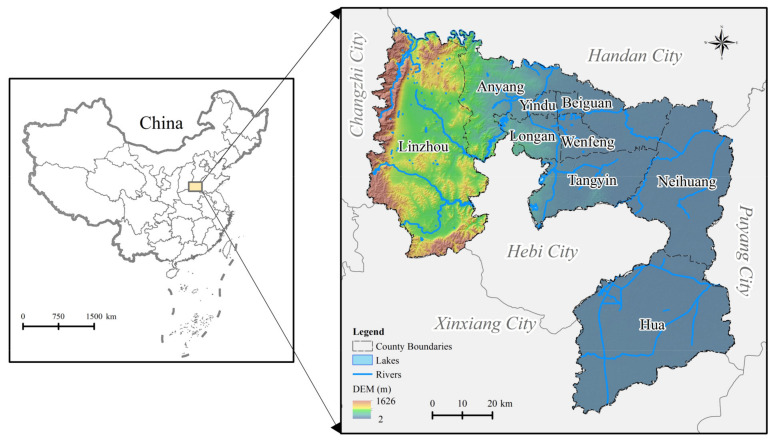
Location map of Anyang.

Anyang (113°38′ E~114°59′ E, 35°41′ N~36°21′ N) is located in the northernmost part of Henan Province in the transitional zone between the North China Plain and the Taihang Mountains (Figure 1). The city has jurisdiction over five counties and four districts with a total area of 7413 km^2^. Anyang has high terrain in the west and low terrain in the east and is spread out in a ladder-like shape, including mountains, hills, plains, depressions, and other land forms, with an altitude range of 2 m to 1626 m. The rivers of Anyang are divided into Haihe River and the Yellow River, with the Jindi River serving as the boundary. The city faces a continental monsoon climate with an average annual temperature of 12.7 °C to 13.7 °C and an annual rainfall of 581.1 mm to 693.1 mm (http://www.anyang.gov.cn/index.jsp (accessed on 3 December 2020)).

As the core area of grain production in the Central Plains economic region, Anyang was planned by the Ministry of Agriculture as a key planting area of grain, cotton, and oilseeds. In 2018, the grain planting area in Anyang reached 5965.33 km^2^, whereas its total grain output reached 3.752 million tons. Anyang is also among those cities in Henan Province with the most important mineral resources and has cultivated an industrial system with pillars, such as metallurgy and building materials, coal chemical industry, equipment manufacturing, textiles, electronic information, and new energy. By the end of 2018, Anyang has posted a GDP of 239.32 billion yuan (6.7% higher than the previous year), which was the seventh highest amount in the province. Based on the statistical yearbook in 2009, Anyang has a population of 5.9227 million, of which 5.176 million are permanent residents, and an urbanization rate of 51.75%.

### 2.2. Data Sources

The basic data used in this research include remote sensing data, socioeconomic statistics, and spatial data (see Table 1 for specific data descriptions). The remote sensing image data were interpreted by using supervised classification and man–machine interactive methods and were verified via field selection, resident interviews, and Google Earth high-resolution remote sensing images [36]. The overall accuracy of these data was over 85%. Based on these data, the land use types in Anyang were divided into cultivated land, woodland, grassland, water area, built land, and unused land. To allow calculation, the scope and format of these data were unified as a 30 m × 30 m raster, and the vector data were registered based on the current land use map and were converted into a raster. Meanwhile, the DEM (Digital Elevation Model) data were processed via projection transformation and resampling. The Euclidean distance was used to calculate the distances from different places to town centers, highways, arterial roads, and railways.

### 2.3. Research Framework

**Figure 2:**
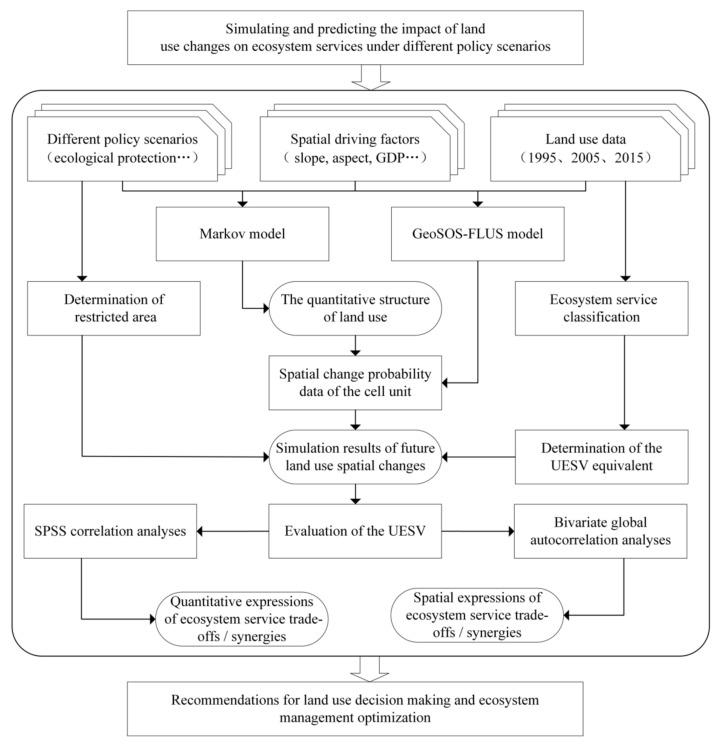
Research framework for simulating and predicting the effects of land use changes on ecosystem services under different policy scenarios. UESV: Ecosystem service value of per unit area.

**Figure 3:**
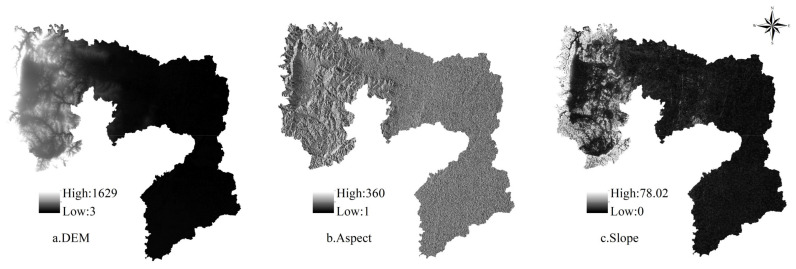

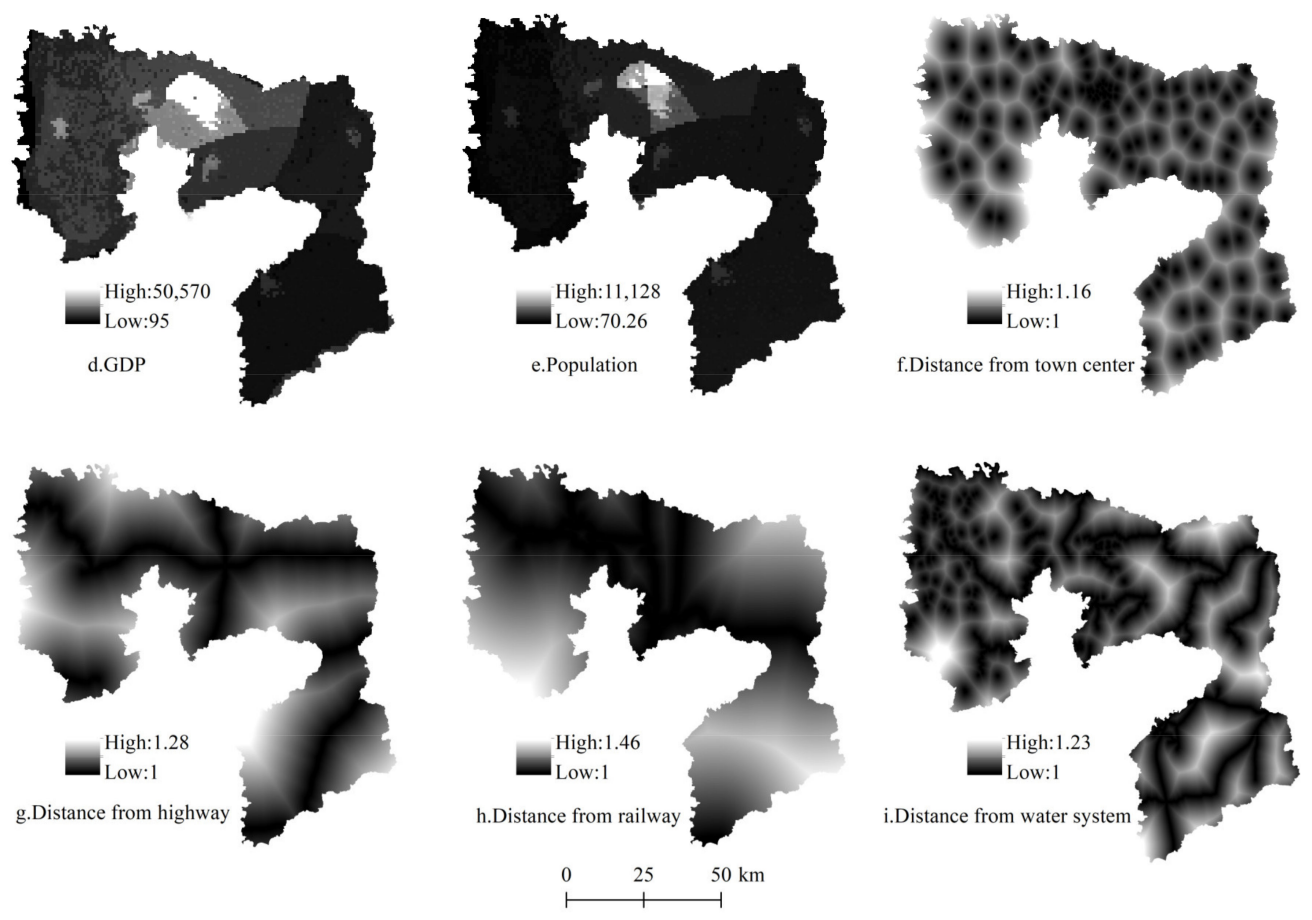
Spatial driving factors of land use simulation.

**Figure 4:**
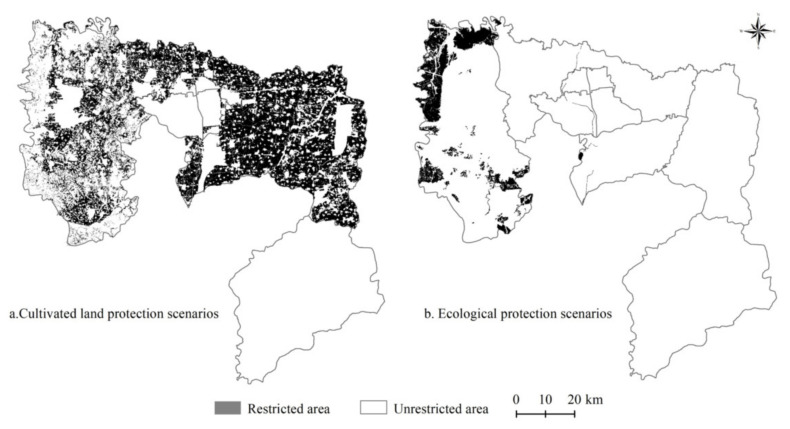
Constraints conditions under different scenarios.

The proposed framework formulates policy recommendations for future land use management and ecosystem services optimization by simulating and predicting the impact of land use changes on ecosystem services under different policy scenarios (Figure 2). This framework was divided into four steps. First, according to different emphases on Anyang’s development mode, three development scenarios, namely, natural evolution, cultivated land protection, and ecological protection, were identified, and based on the constraints of macro-planning control and sustainable land use, the quantitative structure of land use under these scenarios was obtained by applying the Markov model. Second, by using the cellular automata simulation function in GeoSOS-FLUS, spatial driving factors, including digital elevation model (DEM), slope, aspect, GDP, population, distance from town center, distance from highway, distance from railway, and distance from water system, in the research area (Figure 3) were selected to determine the spatial change probability data of the cell unit that combines constraints under different policy scenarios (Figure 4), and the simulation results of future land use spatial changes in Anyang were obtained. Third, based on the land use change prediction results obtained from the simulation and combined with the ESV per unit area (UESV) of Anyang, a simulated prediction of the future ESV of Anyang was realized, and the ecosystem service trade-off and synergy relationship were quantitatively and spatially expressed via correlation analyses and bivariate global autocorrelation analyses. Fourth, after combining the relationship between land use change and ecosystem services under different policy scenarios, recommendations for land use decision making and ecosystem management were proposed.

### 2.4. Methods

#### 2.4.1. GeoSOS-FLUS Model

GeoSOS-FLUS was developed and improved by Li Xia et al. [37], based on the cellular automata (CA) principle. This model can be applied to simulate future land use change scenarios and is generally effective for geospatial simulation, spatial optimization, and decision making. GeoSOS-FLUS initially applies the artificial neural network (ANN) algorithm to estimate land conversion probability based on the driving factors of land use change, combines the land conversion probability, the interaction among cells, and the land change trends to calculate the overall probability of cell transformation, and then couples the Markov and CA models to improve the applicability and realize the simulation of land use change. GeoSOS-FLUS has an adaptive inertial competition mechanism based on roulette selection that can effectively cope with the uncertainty and complexity of the mutual transformation of various land use types under the joint influence of natural and human activities, thereby increasing its simulation accuracy and producing results that closely reflect actual land use distributions [38,39]. The main calculation modules are described as follows:

(1) Calculation of suitability probability based on neural network

ANN includes a prediction and training stage and comprises an input layer, a hidden layer, and an output layer. The suitability probability is calculated as:

(1) $$\begin{array}{ll} sp(p,k,t) & =\sum_{j}\omega_{j,k}\times sigmoid\left(net_{j}(p,t)\right)\\ & =\sum_{j}\omega_{j,k}\times \frac{1}{1+e^{-net_{j}(p,t)}} \end{array}$$

where *p* (*k*, *t*, *l*) represents the suitability probability of the *t* type of land on raster *k* at time *l*, *ω_j_*_,*k*_, *t*, and *sigmoid* are the weight and excitation functions between the hidden and output layers, and *net_j_* (*p*, *t*) is the signal received by the *j* hidden layer grid *p* at time *t*:

(2) $$\sum_{k}sq(p,k,t)=1$$

where the suitability probability *p* (*k*, *t*, *l*) indicates that the sum of all suitability probabilities is 1 on grid *k* at time *l*.

(2) Adaptive inertial competition mechanism

The probability of land use conversion not only depends on the distribution probability of the neural network output but is also affected by certain factors, such as neighborhood density, inertia coefficient, conversion cost, and land competition. The gap between the current land quantity and land demand will be adjusted in an iterative process that determines the inertia coefficients of different land types. The adaptive inertia coefficient of the k land category at time *t* is:

(3) $$Intertia_{k}^{t}\left\{\begin{array}{ll} Intertia_{k}^{t-1} & \left|D_{k}^{t-2}\right|\leq \left|D_{k}^{t-1}\right|\\ Intertia_{k}^{t-1}\times \frac{D_{k}^{t-2}}{D_{k}^{t-1}} & 0\geq D_{k}^{t-2}\geq D_{k}^{t-1}\\ Intertia_{k}^{t-1}\times \frac{D_{k}^{t-1}}{D_{k}^{t-2}} & D_{k}^{t-1}\geq D_{k}^{t-2}\geq 0 \end{array}\right.$$

where $D_{k}^{t-1}$ and $D_{k}^{t-2}$ are the differences between the demand and the number of grids in the *k* type of land at *t* − 1 and *t* − 2, respectively.

After calculating the probability of different grids, the CA model was used to determine the land types. At time *t*, the probability of transforming grid *p* into *k* land type can be expressed as

(4) $$T\mathrm{Pr}ob_{p,k}^{t}=sp(p,k,t)\times \Omega_{p,t}^{t}\times Intertia_{k}^{t}\times (1-sc_{c\to k})$$

where *sc_c_*_→*k*_ is the cost of changing land type *c* to *k*, 1 − *sc_c_*_→*k*_ is the degree of difficulty in the conversion, and $\Omega_{p,t}^{t}$ is the neighborhood function, which is computed as:

(5) $$\Omega_{p,t}^{t}=\frac{\sum {}_{N\times N}con(c_{p}^{t-1}=k)}{N\times N-1}\times \omega_{k}$$

where $\sum {}_{N\times N}con(c_{p}^{t-1}=k)$ denotes the total number of grids of the *k* land after the last iteration in the Moore neighboring window of *N* × *N*. In this paper, *N* = 3, and *ω_k_* denotes the neighboring effect weight of various land types.

The accuracy of the model was verified by observing three parameters, namely, OA, ROC, and Kappa. Parameter values closer to 1 indicate a higher accuracy. Previous studies have shown that the simulation accuracy of GeoSOS-FLUS is higher than that of commonly used models, such as CLUE-S and ANN-CA [37]. Therefore, this model was used in this study to simulate and predict the future ESV of Anyang under different scenarios.

#### 2.4.2. Ecosystem Service Classification and Value Evaluation

Costanza et al. [1] divided terrestrial/marine ecosystem into different ecosystem types and proposed an ecosystem service value evaluation method, but the method has shortcomings when directly applied in China [40]. Based on previous studies [41,42] and combined with the actual situation of Anyang, this study divided Anyang’s ecosystem into six types, namely, cultivated land, woodland, grassland, water, built land, and unused land. The classification standards for the ecosystem service types are not yet unified. By combining the findings of de Groot et al. [3] and the millennium ecosystem assessment (MA) [4] and by considering the previous findings on land type classification and the characteristics of Anyang’s ecosystems, the ecosystem services were further divided into provisioning services (including food production, water supply, and raw material production), regulating services (including gas regulation, climate regulation, hydrological regulation, and environmental purification), supporting services (including soil conservation, maintenance of nutrient cycling, and maintenance of biodiversity), and cultural services (aesthetic landscape). ESV was computed as follows based on the ESV assessment system:

(6) $$ESV=\sum (A_{k}V_{ck})$$

where *ESV* is the total ecosystem service value of the study area (yuan), *A_K_* is the LUCC area of the *k*-type ecosystem (m^2^), and *V_ck_* is the unit price of *k*-type ecosystem service value (yuan/m^2^).

To analyze the differences in the effects of land use changes on ESV within the region, the grid was selected as the evaluation unit. With reference to the grid construction literature and combined with the size of the study area, results show that the commonly used grid analysis units mainly include 500 m × 500 m [43], 1 km × 1 km [44], 2 km × 2 km [45], 3 km × 3 km [46], and 5 km × 5 km [47]. Given the influence of the modifiable areal unit problem on the results, this study compared the spatial and temporal patterns of ESV under different grid units and found that the 1 km × 1 km grid scale can highlight the spatial differences of ESV in Anyang. Therefore, after adjusting the grid size several times, the 1 km × 1 km grid was selected as the basic research unit. The UESV based on traditional ESV was added to eliminate the influence of the differences in the size of the irregular grid at the edge of the study area and to facilitate the comparison. By using the natural breakpoint method, UESV was divided into one to five levels, with a larger UESV corresponding to a higher level [48]. UESV was calculated as follows:

(7) UESV=ESV/S

where *UESV* is the ecosystem service value of the grid (yuan), and *S* is the area of the grid unit (hm^2^).

#### 2.4.3. Determination of the Equivalent UESV of Anyang

The “China Terrestrial Ecosystem Service Value Equivalent Table” proposed by Xie et al. [40] was based on the national average level and was applied on the 1:1 million scale. However, the equivalent value of built land was not defined [49]. Therefore, the ESV at the city scale should be evaluated based on the economic value of food production per unit area of farmland in the region [50].

The economic value of grain production output per unit area of farmland can be determined by using the sown area of main grain crops (including grains, beans, and sweet potatoes in the case of Anyang), their yield, and their national average price (Table 2). This value can be calculated as [51]:

(8) $$E=\frac{1}{7}\sum_{i=1}^{n}\frac{m_{i}p_{i}q_{i}}{M}(i=1,\ldots ,n)$$

where *E* is the economic value of providing food production services per unit area of the cultivated land ecosystem (yuan/hm^2^), *m_i_* is the sown area of *i* food crops (hm^2^), *p_i_* is the national average price of *i* food crops in 2015 (yuan/ton), *q_i_* is the yield per unit of *i* grain crops (t/hm^2^), *M* is the total sown area of grain crops, *n* is the grain type, and 1/7 means that the economic value provided by the natural ecosystem without human input is 1/7 of the economic value of food production services provided by the unit area of farmland [52].

According to Formula (7), the economic value of annual grain output per unit area of farmland in Anyang was 127.32 yuan/hm^2^. The evaluation coefficient for the ESV of Anyang was then calculated as shown in Table 3.

#### 2.4.4. Quantitative and Spatial Explicit of Ecosystem Service Trade-Offs and Synergies

An ecosystem provides more than one type of service to the society. If different ecosystem services depend on the same ecological process or are affected by specific external factors at the same time, they may interact with one another and produce dynamic changes, such as trade-offs and synergies [53]. Mastering the types of trade-offs and synergies among these services serves as a foundation and prerequisite for a sustainable ecosystem service management [54]. The available methods for measuring the trade-offs and synergies of ecosystem services include the ecological–economic integrated model [55], correlation analysis [56], spatial analysis mapping methods [57], and scenario simulation [58]. Correlation analysis is a simple and effective method for identifying the type and degree of ecosystem service trade-offs and synergies; accordingly, this approach has been widely used by scholars [59,60,61]. Spatial autocorrelation analysis was then performed in this study to measure the trade-offs and synergies of ecosystem services.

Spatial autocorrelation analysis mainly measures the correlation and spatial heterogeneity among geographic entities and can be applied at the global and local levels [62,63]. The former tests and determines the overall spatial distribution pattern and the degree of correlation and significance of elements in the research area, whereas the latter reflects the agglomeration and differentiation characteristics of local spatial units and their neighboring units to further measure local spatial instability. The bivariate spatial autocorrelation analysis was further improved based on these two levels and can be used to measure the degree of spatial correlation between the attributes of two variables [64]. The bivariate global and local autocorrelation models can be formulated as:

(9) $$I=\frac{\left[n\sum_{i=1}^{n}\sum_{j-1}^{n}W_{ij}(y_{i}^{m}-y_{m})(y_{j}^{z}-y_{z})\right]}{(\sum_{i=1}^{n}\sum_{j-1}^{n}W_{ij}\sum_{i=1}^{n})(y_{i}^{m}-y_{m})(y_{j}^{z}-y_{z})}$$

(10) $$I_{ij}=Q_{i}^{m}\sum_{j=1}^{n}(W_{ij}Q_{j}^{z});Q_{i}^{m}=\frac{y_{i}^{m}-y_{m}}{\sigma_{m}};Q_{j}^{z}=\frac{y_{j}^{m}-y_{z}}{\sigma_{z}}$$

where *I* is the global bivariate spatial autocorrelation index, *n* is the number of grid cells, *W_ij_* is the spatial weight, $y_{i}^{m}$ and $y_{j}^{z}$ are the *m* and *z* attribute values of the *i* and *j* grid cells, respectively, *y_m_* and *y_z_* are the average values of attributes *m* and *n*, respectively, *I_ij_* is the local bivariate spatial autocorrelation index, and *σ_m_* and *σ_z_* are the variances of attributes *m* and *z*, respectively. *I* takes a value within the range of [–1, 1], which represents the trade-off and synergy among provisioning, regulating, supporting, and cultural services. When the value of *I* is greater than 0 and closer to 1, the synergy between ecosystem services becomes more significant. When *I* is 0, no trade-off or synergy relationship is observed. When *I* is less than 0 and closer to –1, the trade-off relationship becomes highly obvious. The *I_ij_* value was calculated by using the Geoda software to obtain the local indicators of spatial association (LISA) cluster map and to analyze the local spatial autocorrelation pattern of the trade-off and synergy among different ecosystem services. The high–high and low–low agglomerations indicate a synergy relationship, whereas the high–low and low–high agglomerations indicate a trade-off relationship.

## 3. Results

### 3.1. Land Use Change and Scenarios Simulation in Anyang

**Figure 5:**
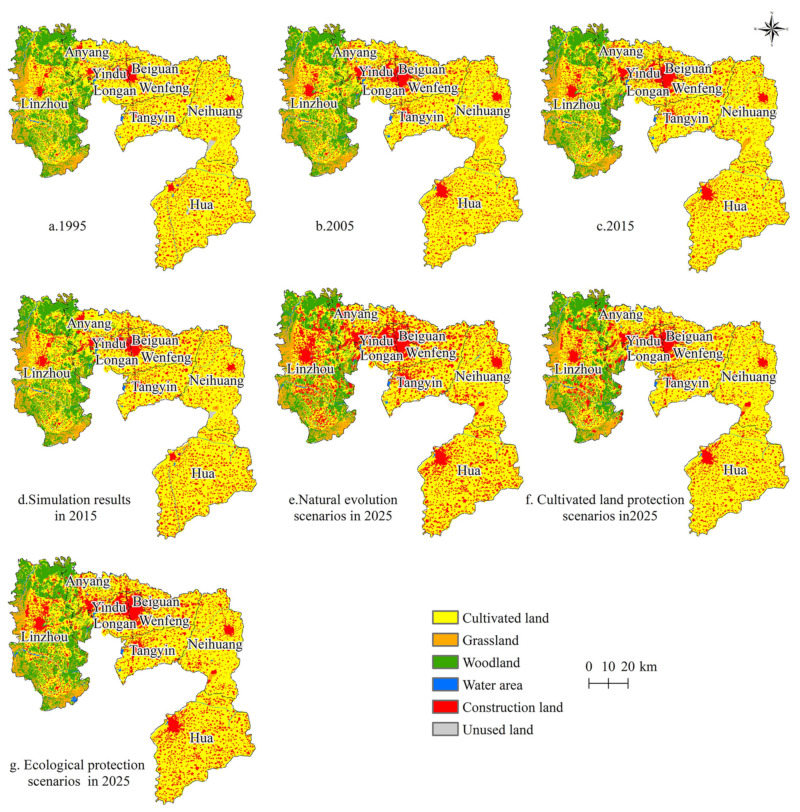
Spatio-temporal evolution and predictions of land use in Anyang from 1995 to 2025.

**Figure 6:**
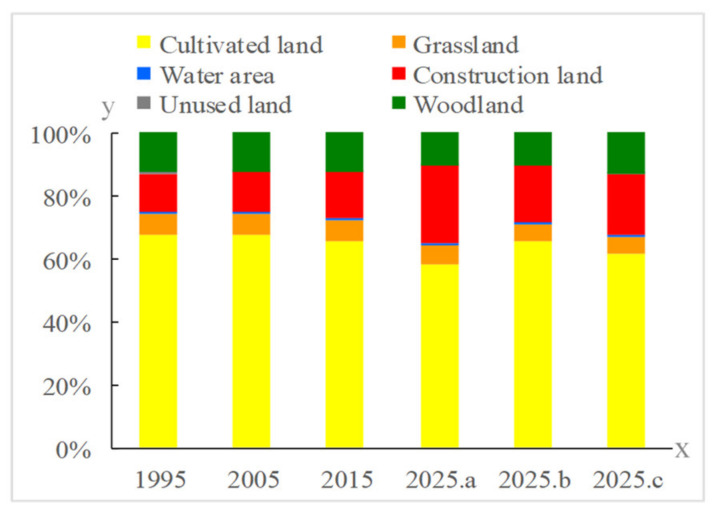
Changes in the land use structure of Anyang from 1995 to 2025 (Where a, b, and c indicate the natural evolution, cultivated land protection, and ecological protection scenarios, respectively).

To predict the land use changes in Anyang under different scenarios in 2025, the data for 2005 were used to simulate the land use status for 2015, and the simulation results were compared with the actual land use data for 2015. The Kappa value was 0.72, whereas the Fom value was 0.0643, both of which met the accuracy requirements. Afterward, based on the Markov model, the current status data for 2005 and 2015 were used to estimate future land use demand and to predict the land use situation in Anyang under different scenarios in 2025 (Figure 5 and Figure 6).

From 1995 to 2015, the land use changes in the built and cultivated lands of Anyang were the most dominant, with built land showing the most obvious increase in size (21,258.86 hm^2^). Meanwhile, the cultivated land area showed the most obvious decrease in size (15,609.55 hm^2^) primarily due to the rapid social and economic development of Anyang since the 1990s. The urbanization rate of Anyang increased from 13.64% in 1995 to 52.3% in 2015, which led to the rapid expansion of urban construction and rural residential lands and the occupation of a large amount of cultivated fields. The spatial distribution patterns indicate that the main land use types in Anyang from 1995 to 2015 include cultivated land, woodland, and built land, among which cultivated land was mainly distributed in the east and south, woodland was mainly distributed in the east, and built land was mainly distributed in the center and east. These distribution patterns are limited by the special geographical environment of Anyang, which is surrounded by the eastern foot of Taihang Mountain to the west and the Huanghuaihai Plain to the east. In addition, Anyang has as high terrain in the west and low terrain in the east.

From 2015 to 2025, the general development model revealed a sharp increase in built land (181,088.71 hm^2^) as a result of natural evolution, and this land type occupied a large amount of cultivated land and woodland. Meanwhile, the spatial distribution patterns suggest that the increase in built land was mainly concentrated in Anyang and Linzhou. This trend is consistent with the upgrading of Anyang County to a sub-center of the city and the proclamation of Linzhou as an important node of the urban development axis in Northern Henan. Under the cultivated land protection scenario, the built land area was largely reduced to 132,731.98 hm^2^. While the areas of cultivated land, garden land, woodland, water, and unused land also decreased, such decline was not as significant as that reported in 2015. From the spatial layout, urban land showed a compact development, thereby reflecting the spatial control effect of basic farmland on urban land development, which is conducive to the formation of a forceful mechanism for improving land use efficiency. Under the ecological protection scenario, the areas of woodland and built land significantly increased to 94,994.68 hm^2^ and 36,675.43 hm^2^, respectively, following the transfer of cultivated land and garden land. From the spatial layout, the built land in central urban areas slightly increased, whereas the woodland in Linzhou significantly increased, thereby suggesting that the implementation of ecological protection policies curbs the expansion of built land.

### 3.2. ESV Change and Scenarios Simulation in Anyang

**Figure 7:**
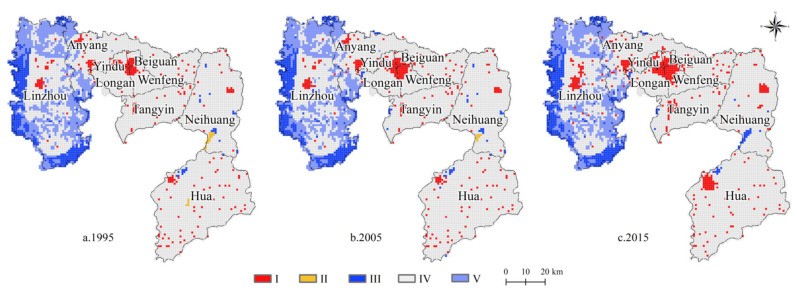
Spatial-temporal evolution of UESV in Anyang from 1995 to 2015. I: <−3.3 billion yuan. II: −3.3~0 billion yuan. III: 0~1.3 billion yuan. IV: 1.3~3.5 billion yuan. V: >3.5 billion yuan.

From the perspective of the spatial distribution pattern (Figure 7), the UESV of Anyang showed some significant regional differences across three periods, with high spatial patterns in the west, north, and south and low spatial patterns in the east and center. The high UESV area in the west had a high altitude and was mostly comprised of woodland, thereby explaining its favorable natural ecological background conditions. Meanwhile, the low UESV areas in the east and center had a flat terrain and were mainly comprised of built and cultivated land with low ESVs. From 1995 to 2015, the spatial coverage of low UESV areas in Anyang continued to expand, whereas that of high UESV areas continuously shrank. This trend is consistent with the increasing built land in Anyang as a result of its expansion to cultivated land, woodland, and grassland.

**Figure 8:**
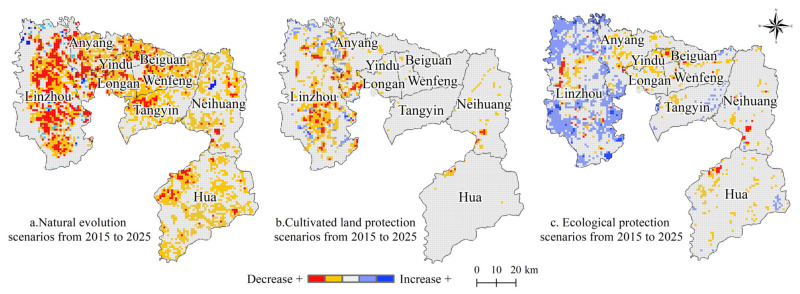
Changes in the ESV of Anyang from 2015 to 2025.

From 2015 to 2025, the overall change in UESV across different scenarios was significant (Figure 8), with the natural evolution scenario showing the most significant decline, followed by the cultivated land and ecological protection scenarios. From the perspective of the internal space grid, the grid UESV under the natural evolution scenario showed a decline, especially in the west, mainly due to the small policy constraints and economic benefits that brought great pressure to its ecological environment. Under the cultivated land protection scenario, the grid UESV value did not change significantly. Under the influence of a strict cultivated land protection policy, the occupancy rate of cultivated land decreased, and the expansion of built land was restricted. Therefore, the UESV in most areas tended to stabilize, and the cultivated land protection policy also protected the ecology surrounding the cultivated land, hence explaining why the UESV of some areas increased. Under the ecological protection scenario, the UESV in western Anyang significantly increased mainly due to its favorable ecological background conditions and strict ecological protection policies. In the central and eastern areas, the UESV slightly declined as a result of the encroachment of cultivated land by built land.

**Figure 9:**
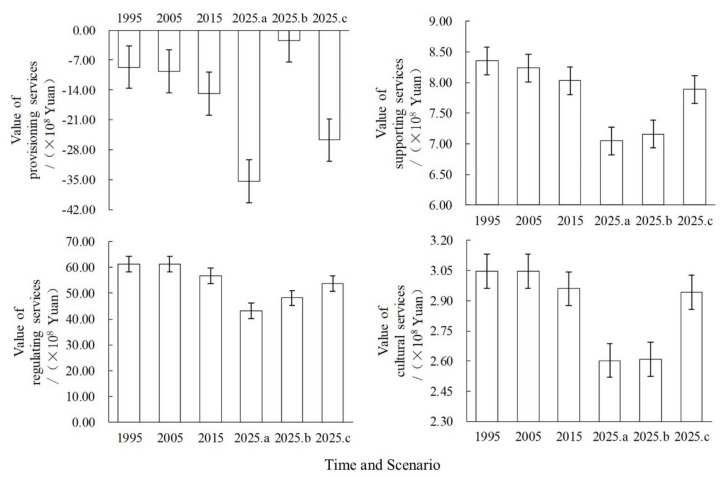
Provisioning, regulating, supporting, and cultural ESV changes from 1995 to 2025 (Where a, b, and c indicate the natural evolution, cultivated land protection, and ecological protection scenarios, respectively).

Looking at the ESV of different service types (Figure 9), the value of provisioning services in Anyang showed a decline from 1995 to 2015, amounting to a loss of 623 million yuan. Under the three scenarios from 2015 to 2025, the provisioning service value under the natural evolution and ecological protection scenarios decreased by 2.053 and 1.083 billion yuan, whereas that under the cultivated land protection scenario slightly increased to 1.251 billion yuan. This can be ascribed to the fact that the former two scenarios failed to attach importance to the protection of cultivated land, thereby reducing the provisioning service value, whereas the cultivated land protection scenario can effectively reduce the encroachment of cultivated land and increase the provisioning service value (e.g., food production). From 1995 to 2015, the value of regulating service showed a decline with losses of 472 million yuan. However, from 2015 to 2025, the value of regulating services slightly decreased under the three scenarios. The value of services in the natural evolution scenario decreased the most mainly due to the imbalanced land use structure that resulted in a significant decline in the value of regulating services, such as climate and hydrological regulation. From 1995 to 2015, the value of Anyang’s supporting services showed a decline with losses of 33 million yuan. Similarly, this value decreased from 2015 to 2025, with the ecological protection scenario showing the slightest decrease of only 14 million yuan, thereby indicating that ecological land protection can effectively control the decline in service value. From 1995 to 2015, the value of culture services showed a decline with a decrease of 9 million yuan. A similar decline was observed from 2015 to 2025, with the natural evolution and cultivated land protection scenarios showing the greatest declines of 36 and 35 million yuan, respectively. Meanwhile, the value of culture services under the ecological protection scenario was stable, thereby indicating that the woodland and grassland were strictly controlled under this scenario and that the aesthetic landscape service was effectively developed.

### 3.3. Trade-Offs and Synergies among Different Ecosystem Services Across Different Scenarios

To explore the trade-offs and synergies among different types of ecosystem services under various policy scenarios in Anyang, a 1 km grid unit was used as the evaluation unit to obtain the UESV of a single service for each grid unit across all scenarios, followed by correlation analyses and bivariate global autocorrelation analyses. The Pearson correlation coefficient was greater than 0, thereby suggesting that different ecosystem services were synergistic. A larger coefficient indicates that the synergy of ecosystem services becomes more significant over time. By contrast, the relationship was a trade-off. From the perspective of the Pearson correlation coefficient and the bivariate global autocorrelation Moran’s I index under the three scenarios, the provisioning, regulating, supporting, and cultural services of Anyang were consistent. The synergy relationship was most significant yet negative under the natural evolution scenario, thereby suggesting that the value of most ecosystem services was synergistically reduced. In terms of Pearson coefficient, the correlation coefficients between ecosystem services were ranked as p-r (provisioning service-regulating service) > s-c (supporting service-cultural service) > r-c (regulating service-cultural service) > p-s (provisioning service-supporting service) > p-c (provisioning service-cultural service) > r-s (regulating service-supporting service). Meanwhile, in terms of Moran’s I index, the correlation coefficients between ecosystem services were ranked as p-r > s-c > r-c > p-c > p-s > r-s (Table 4).

**Figure 10:**
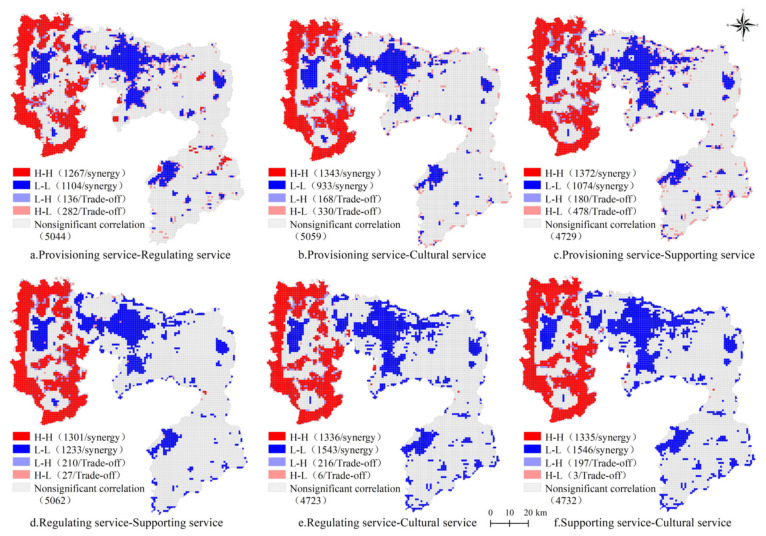
Local LISA of four ecosystem services under the natural evolution scenario in Anyang in 2025. H-H: high–high agglomeration; L-L: low–low agglomeration; L-H: Low–high agglomeration; H-L: high–low agglomeration (the same below).

**Figure 11:**
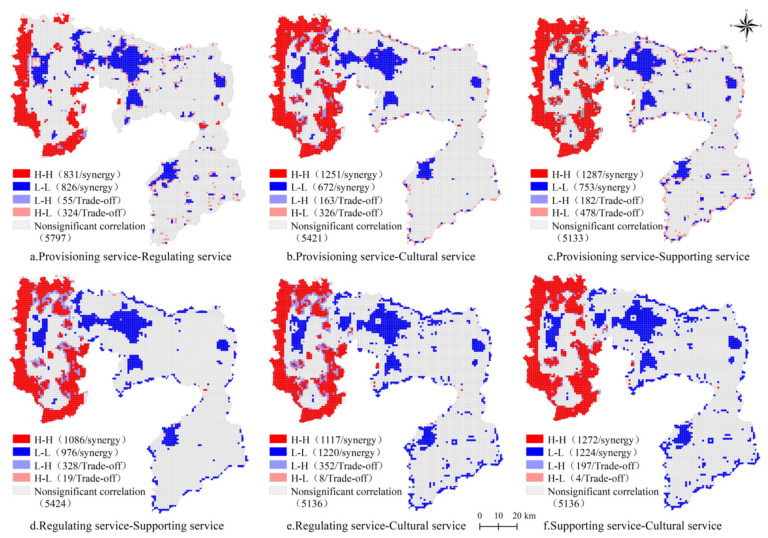
Local LISA of four ecosystem services under the cultivated land protection scenario in Anyang in 2025.

**Figure 12:**
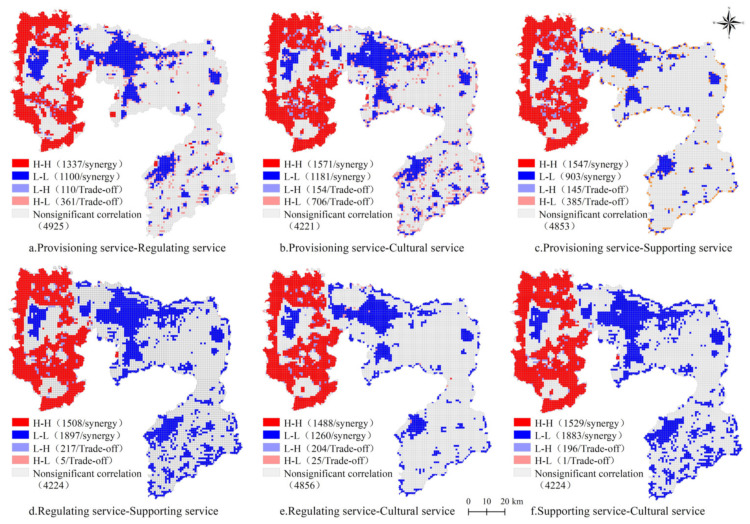
Local LISA of four ecosystem services under the ecological protection scenario in Anyang in 2025.

To further understand the synergy and trade-off relationship among different ecosystem services in Anyang, a bivariate local autocorrelation analysis was performed. The spatial agglomeration characteristics of the synergy and trade-off relationship among the ecosystem services under the three scenarios were significant (Figure 10, Figure 11 and Figure 12). The main spatial relationship among provisioning, regulating, supporting, and cultural services was synergy, distributed in the eastern and central areas of Anyang, and partially showed a trade-off relationship that was distributed in Linzhou, Huaxian, and the surrounding areas of Anyang. The number of grids where p-r, p-c, p-s, r-c, r-s, and s-c showed a synergistic relationship under the natural evolution scenario were 2371, 2276, 2446, 2534, 2879, and 2881, respectively, and the number of grids showing a trade-off relationship were 418, 498, 658, 237, 222, and 200. Meanwhile, the number of grids where p-r, p-c, p-s, r-c, r-s, and s-c show a synergistic relationship under the cultivated land protection scenario were 1657, 1923, 2040, 2062, 2337, and 2496, respectively, and the number of grids showing a trade-off relationship were 379, 489, 660, 347, 360, and 201. The number of grids where p-r, p-c, p-s, r-c, r-s, and s-c show a synergistic relationship under the ecological protection scenario were 2437, 2752, 2450, 3405, 2748, and 3412, respectively, whereas the number of grids showing a trade-off relationship were 471, 860, 530, 222, 229, and 197. The synergy under the three scenarios in western Anyang was manifested as high-high agglomeration due to the large amount of woodland and the high ecological background quality in the west. However, the synergy in the central part was manifested as low-low agglomeration mainly due to the large number of built land and the rapid outward expansion of the city. Under these three scenarios, the grids showing trade-off relationships were scattered across various districts, most of which were in the periphery of ecological lands, such as woodland, or among the built land, woodland, and cultivated land. Given that economic development had destroyed the stable ecosystem structure, a trade-off feature was observed.

## 4. Discussion

### 4.1. Framework of Simulating Effects of Land Use Changes on Ecosystem Services

Ecosystem services, which bring various benefits to humans, reflect the direct and indirect use of land resources by mankind and are largely influenced by changes in land use patterns. Changes in land use can lead to variations in ecosystem services, and the status of ecosystem services can also reflect the status of land use. Although some studies have used future land use changes to predict ESV [65,66], only few have predicted and analyzed the trade-off and synergy among ecosystem services under different future scenarios. The existing land use simulation models mainly include the CA-Markov model [67], SD model [68], CLUE-S-model [69], SLEUTH model [70], and Tietenberg model [71]. However, most of these models are unable to analyze the relationship among different land types, thereby introducing challenges in characterizing the competition and interaction among these land types under an increasing urbanization. The GeoSOS-FLUS model based on an adaptive inertial competition mechanism can solve these problems effectively [37], but this model has been rarely applied in predicting ESV and analyzing the trade-offs and synergy among future ecosystem services. The proposed framework based on GeoSOS-FLUS (Figure 2) demonstrates great improvements over those developed in previous studies. First, this framework can measure the ESV under different policy scenarios and simulate the impact of land use change on ecosystem services under multiple scenarios. Second, this framework can explore the relationship among different ecosystem services under different policy scenarios and simulate the impact of land use change on the trade-off or synergy of these services.

According to the proposed framework, land use simulation show that compared with 2015, the area of built land rapidly increased in 2025, whereas the area of cultivated land sharply decreased. The ESV under three land use scenarios was then calculated based on land use forecast data for 2025 and the UESV in Anyang (Figure 8). Results show that the total ESV in 2025 suffered from varying degrees of losses under different land use scenarios, with the natural evolution scenario incurring the largest loss of 3.536 billion yuan, followed by the cultivated land protection scenario (1553 billion yuan) and the under ecological protection scenario (1.308 billion yuan), thereby suggesting that the cultivated land and ecological protection scenarios can alleviate the decline in ESV under the natural scenario, which is consistent with the findings of Li et al. [72] and Wang et al. [73]. In addition, the built land under the natural evolution scenario was expanded by filling and was mainly distributed around the built-up area, along lakes and rivers, and along the road traffic network. The built land under the cultivated land and ecological protection scenarios showed different degrees of concentration and contiguous space. Restricted by the ecological red line and the basic farmland protection area, the built land under these scenarios was more compact than the expansion layout observed under the natural evolution scenario and was mainly distributed around the urban area of Anyang. The land area was small yet expands at a stable speed.

Based on the proposed framework, the analysis of trade-offs and synergies reveals that the overall relationships between ecosystem services under the three scenarios in 2025 tend to be the same. Therefore, the internal spatial heterogeneity should be analyzed further to formulate ecological protection policies according to the local conditions. The partial trade-off relationship revealed a competition in regional land use to a certain extent and some conflicts among economic development, food production, and ecological protection. Moreover, results on the coordination relationship among regulating, supporting, and cultural services are consistent with those of previous studies [74], but the relationship between provisioning services and other services was synergized, thereby contradicting the findings of some scholars [75]. Such a discrepancy can be ascribed to the significant spatial heterogeneity of the trade-offs and synergies between ecosystem services. Each region has unique different trade-offs and synergies, and for those ecosystem services that fluctuate from one region to another, some synergistic relationships may also be observed.

### 4.2. Policy Enlightenments

Land use changes are spatially different, and the variations in ESV under the influence of these changes also show obvious spatial differences. Therefore, implementing an undifferentiated land use optimization policy in the entire region will not be able to protect and enhance the regional ESV. Therefore, this study analyzed the impact of future land use changes on ESV and performed a targeted spatial control of land use to improve regional ESV with respect to the local conditions. There are two main spatial regions where the land use changes in Anyang will greatly affect ESV. The first is control area or land for urban construction. Anyang is a regional central city located at the junction of Henan, Shanxi, and Hebei that shows a coordinated development around Beijing, Tianjin, and Hebei. As a result of rapid urbanization, the expansion of urban built land encroaches a large amount of cultivated land, woodland, and water, thereby reducing the ESV of areas around towns. From the perspective of land use trend, the current urban built land continues to expand, which may introduce serious ecological disturbances in the urban fringe and threaten the coordinated and sustainable development of the economy, society, and ecology in Anyang. In the future, the total scale of built land should be controlled to realize the transformation of land use from incremental supply to stock tapping, especially in the central urban area. This approach can effectively improve the ESV of the counties and cities of Anyang. The second is ecological protection area, including woodlands and waters. Linzhou is located at the eastern foot of the Taihang Mountain, and mountains and hills account for 86% of its total area. Linzhou is rich in forest resources and has four natural rivers (Qihe River, Huan River, Xihe River, and Zhanghe River) and an artificial river (Red Flag Canal) that account for its water resources of 565.2 million cubic meters. Woodland and water are land types with high ecological value coefficients, thereby explaining the formation of high ESV areas in Linzhou. However, when lands with high ESV, such as woodland and water, are destroyed, the original ecological system cannot be easily rebuilt. Therefore, protecting the woodland and water should be prioritized in future land use planning. On the one hand, protecting these land types, identifying the balance point among population, economy, resources, and environment, and controlling the population scale, industrial structure, and growth rate within the available resource and environmental capacities are necessary. On the other hand, the advantages of ecological resources, such as woodland and water bodies, should be used to develop tourist cities and natural oxygen bars, to cultivate high-efficiency economic forests, to increase people’s income, and to formulate appropriate ecological compensation mechanisms for ensuring the sustainable development of the ecological economy.

### 4.3. Methodological Concerns

The currently available methods for evaluating ESV include the market alternative method [76], shadow price method [77], and opportunity cost method [78], among which the market alternative method also known as the value equivalent method [40], is widely used. Value equivalent factors are essentially the equivalent coefficients that are obtained via a comprehensive scoring system developed by a large number of ecosystem research experts. These equivalent coefficients have been revised according to the actual situation in China and are more suited to China’s national conditions than those indicators proposed by Costanza et al. [41]. The equivalent factor method can also rapidly obtain and calculate data, has strong applicability, and can facilitate the comparison of results [64]. Therefore, this approach was adapted in this paper based on the actual situation in Anyang. Results show that the ESVs of Anyang in 1995, 2005, and 2015 amounted to 6.401, 6.283, and 5.275 billion yuan, respectively, showing a declining trend that is consistent with the findings of previous research. Therefore, this method can efficiently predict the changes in the ESV of Anyang. However, the complexity, dynamics, and non-linear characteristics of the ecosystem may introduce defects into the equivalent factor method. First, the differences in the correction methods and parameters for the same research area can lead to differences in the evaluation results, and local differences are difficult to measure with uniform parameters. For example, Xie et al. [41] used the value equivalent factor method to calculate the ESV of China in 2010 and found that Hainan has an ESV of 448.825 billion yuan. Meanwhile, Lei et al. [79] used the revised unit area value equivalent factor method to assess the ESV of Hainan Island from 1980 to 2018 and obtained an ESV ranging from 201.403 to 208.449 billion yuan. Therefore, a more accurate evaluation model should be developed to identify the non-linear change characteristics of ESV and the local specific information at different scales as well as to improve the comprehensiveness and scientific nature of ESV evaluation. Second, the equivalent factor method simply links the classification of land use to the type of the natural ecosystem, which are not completely consistent. Specifically, different types of woodland and grassland are indistinguishable (e.g., woodland can be divided into forest land, shrubs, and sparse woods), thereby limiting this method to making mere approximations of the ESV of various land use types. Future studies should explore better ways of subdividing land use types to accurately correspond to natural ecosystems and investigate how to achieve a more effective ESV estimation.

The correlation coefficient method can directly reveal the numerical relationship of ecosystem service trade-off/synergy. Bivariate spatial autocorrelation can characterize the spatial relationship of ecosystem service trade-off/synergy but cannot fully reflect the internal and action mechanism of ecosystem services. Future studies may employ other methods and means for further discussion and deep analysis. In addition, some scholars [80] believe that the relationship among ecosystem services shows phased and differentiated features over time and argued that studying the relationship among regional long-term series and continuous time periods can improve the reliability of the weighing results. Therefore, future studies should explore the time scale effect of the trade-off/synergy relationship and explore such relationship over the long term.

## 5. Conclusions

Given its accelerating urbanization, the land use pattern of Anyang significantly changed over the years, with built land showing the most significant expansion. Meanwhile, the amount of its woodland, grassland, and cultivated land all showed a decreasing trend, whereas the size of its water remained stable. In 2025, the degree of land use change under the cultivated land and ecological protection scenarios are expected to be more moderate than that under the natural evolution scenario. Human activities and policy intervention would have a significant impact on land use patterns.

The ESV of Anyang generally decreased from 1995 to 2015, with a total reduction of 1126 million yuan. Meanwhile, the declining trend in this ESV from 2015 to 2025 is expected to be more significant under the natural evolution scenario than that under the cultivated land and ecological protection scenarios. The spatial distribution characteristics of UESV in each period were generally consistent. High values were concentrated in the high-altitude forest areas of Linzhou, whereas low values were distributed in the surrounding flat built land. The ESV in the western and central regions of Anyang showed a downward trend and warrant further research.

From 1995 to 2015, the four ESV, including provisioning, regulating, supporting, and cultural services, showed an overall downward trend with a decrease of 623, 472, 33, and 9 million yuan, respectively. From 2015 to 2025, the value of the prat ecosystem services under the three scenarios is expected to decline, but the values under the cultivated land and ecological protection scenarios would only show a slight decline.

The relationships between various ecosystem services in Anyang under different scenarios in 2025 were consistent and showed an obvious synergy, especially under the natural evolution scenario. The agglomerations of “high-high” synergy in the west and “low-low” synergy in the central region were obvious, but the number and distribution of each grid differed. Local areas showed “high-low” and “low-high” trade-off relationships that were scattered in grids among built land, woodland, and cultivated land.

Based on GeoSOS-FLUS, this study constructed a framework for studying the effects of land use change on ecosystem services under different policy scenarios. This framework predicts the future changes in ecosystem services in Anyang and analyzes the trade-offs and synergies among future ecosystem services. Results show that this framework can provide certain scientific support for regulating land use and ecosystem services in rapidly urbanized regions.

## Figures and Tables

**Table 1:** Data sources and usage.

Data Type	Data Content	Data Sources	Data Usage
Remote sensing data	Landsat-TM images in 1995, 2005 and 2015 (30 m × 30 m grid)	Geospatial Data Cloud Platform	Model basic input data
Statistical data	Population data, food production, GDP, etc.	Anyang Statistical Yearbook (1996, 2006 and 2016)	ESV (Ecosystem service value) calculation
Topographic data	Elevation (DEM) (30 m × 30 m grid)	Geospatial Data Cloud Platform	Natural terrain driving force factor
Traffic data	National highways, provincial highways, highways, etc. (vector)	AMAP (AutoNavi map)	Traffic location driving force factor
Basic farmland data	Basic farmland database of Anyang (vector)	Anyang Natural Resources Bureau	Restricted conversion area
Planning data	Anyang Ecological Reserve (vector)	Anyang Natural Resources Bureau	Restricted conversion area

**Table 2:** Sown area, grain yield, and national average unit price of main crops in Anyang.

Crop type	Variety	Sown Area (hm^2^)	Grain Yield (10,000 tons)	Average Unit price (Yuan/t)
Cereals	Wheat	308,968	5.84	2413
	Maize	236,884	5.57	1771
	Sorghum	17,000	3.20	2400
Beans	Soybean	5206	2.64	3529
	Miscellaneous beans	464	1.44	3488
Potatoes	Sweet potatoes	8127	6.59	2000

**Table 3:** ESV coefficient per unit area in Anyang (100 yuan/hm^2^).

Ecosystem Classification		Cultivated Land	Grassland	Woodland	Water	Built Land	Unused Land
Provisioning services	Food production	14.68	10.01	5.35	13.82	0.00	0.00
	Raw materials production	6.91	9.59	12.26	3.97	0.00	0.00
	Water resources supply	0.35	3.37	6.39	143.19	−268.66	0.00
Regulating services	Gas regulation	11.57	26.08	40.59	13.3	−45.6	0.35
	Climate regulation	6.22	63.82	121.43	39.56	0.00	0.00
	Environment purification	1.73	18.05	34.37	95.87	−46.35	1.73
	Hydrology regulation	46.64	32.65	60.63	1766.01	0.00	0.52
Supporting services	Soil maintenance	17.79	33.6	49.4	16.06	0.00	0.35
	Maintaining nutrient circulation	2.07	2.94	3.80	1.21	0.00	0.00
	Biological diversity	2.25	23.58	44.91	44.05	0.00	0.35
Cultural services	Aesthetic landscape	1.04	10.36	19.69	32.65	0.00	0.17

**Table 4:** The correlation for the four ecosystem services across different scenarios in Anyang in 2025.

Category	Pearson Coefficient			Moran’s I		
	Natural Evolution	Cultivated Land Protection	Ecological Protection	Natural Evolution	Cultivated Land Protection	Ecological Protection
*R_ps_*	0.552	0.306	0.519	0.354	0.232	0.346
*R_pc_*	0.511	0.441	0.490	0.358	0.289	0.365
*R_rs_*	0.397	0.334	0.398	0.303	0.235	0.322
*R_rc_*	0.748	0.645	0.710	0.540	0.499	0.533
*R_sc_*	0.826	0.662	0.813	0.580	0.526	0.590
*R_pr_*	0.963	0.963	0.963	0.712	0.705	0.729

Note: *R_ps_*—correlation coefficient between provisioning services and supporting services; *R_pc_*—correlation coefficient between provisioning services and cultural services; *R_rs_*—correlation coefficient between regulating services and supporting services; *R_rc_*—correlation coefficient between regulating services and cultural services; *R_sc_*—correlation coefficient between supporting services and cultural services; *R_pr_*—correlation coefficient between provisioning services and regulating services.

## Data Availability

The data presented in this study are available on request from the corresponding author.
